# Contemporary management of intracardiac thrombi: a tale of two clots

**DOI:** 10.1051/ject/2023010

**Published:** 2023-06-28

**Authors:** Ben Branigan, Sam Brown, Ryan Zavala, HelenMari Merritt

**Affiliations:** 1 Department of General Surgery, Iowa Methodist Medical Center Des Moines IA 50309 USA; 2 Department of Cardiothoracic Surgery, Loma Linda University Loma Linda CA 92354 USA; 3 Innovative ECMO Concepts Omaha NE 68114 USA; 4 Methodist Physicians Clinic Omaha NE 68114 USA

**Keywords:** Thrombosis, Pulmonary Embolus, Circulatory Arrest

## Abstract

Intracardiac thrombi such as pulmonary emboli represent a high risk of mortality. In this case study, we review two cases of intracardiac thrombi occurring within 24 h of one another and managed differently by the same cardiothoracic surgical team, highlighting the importance of an individualized approach as well as an understanding of current guidelines and contemporary management techniques.

## Introduction

Pulmonary embolus (PE) remains the third most common cause of cardiovascular death in the United States [1]. Mortality remains especially high (between 15 and 57%) for patients who present with massive or submassive PE [1]. Massive PE is defined as a pulmonary embolism causing cardiogenic shock and hypotension. Submassive PE is characterized by a pulmonary embolism causing right ventricular dysfunction or elevations in cardiac enzymes without frank cardiogenic shock [2]. Modern technologies afford cardiothoracic teams a variety of modalities for the management of intra-cardiac thrombus, including PE. Treatment for massive or submassive PE typically involves systemic anticoagulation. The addition of interventions focused on directly removing or breaking down the embolus itself may help offload the right ventricle (RV) and avoid hemodynamic collapse. These interventions include systemic thrombolytic drug administration, minimally invasive catheter-based therapies for targeted thrombolysis or embolectomy, or open surgical pulmonary embolectomy [1].

Surgical embolectomy (SE) is considered for patients with massive or submassive PE with at least moderate RV dysfunction and a contraindication to fibrinolytic, which is present in up to 40% of patients [1, 2]. Other indications for a surgical approach include patients with a clot-in-transit or those with an atrial septal defect including a large patent foramen ovale [2]. SE involves urgent sternotomy, arresting the heart with a cardiopulmonary bypass (CPB) circuit, and directly opening the pulmonary arteries with the removal of the thrombus. Overall survival ranges from 67% to 89% and may be related to patient status at the time of surgery, with patients in arrest or those with impending cardiovascular collapse fairing less well [3, 4].

Patients undergoing surgical embolectomy following the failure of lytic therapy have the worst outcomes overall [4].

There are several devices currently available for consideration in patients with massive or submassive PE. The AngioVac (Angiodynamics, Latham, NY) device was approved by the FDA in 2009 for the percutaneous removal of fresh thrombi [5]. Other indications which have been described include debridement of right-sided endocarditis, reduction in thrombus burden in a pulmonary embolus, and utilization for capturing particulate matter in cases where embolic material may be a threat (i.e. indwelling catheter or device-related thrombus or vegetation) [6]. In this approach, an aspiration cannula is inserted into one of the major veins and connected to a veno-venous bypass circuit which then captures the aspirated debris. Although there have been case series that focus on the reduction in thrombus burden and hospital survival, data are lacking on the impact of these devices long-term on survival and patient outcomes [6, 7].

The following two cases occurred within 24 h of one another and illustrate the importance of the ability of the cardiac surgery team to employ both traditional open surgical procedures and contemporary minimally invasive techniques. One case describes an open pulmonary embolectomy, while the other describes the use of the AngioVac system and veno-venous bypass for removal of SVC and Right Atrial Thrombus.

## Case 1

**Figure 1:**
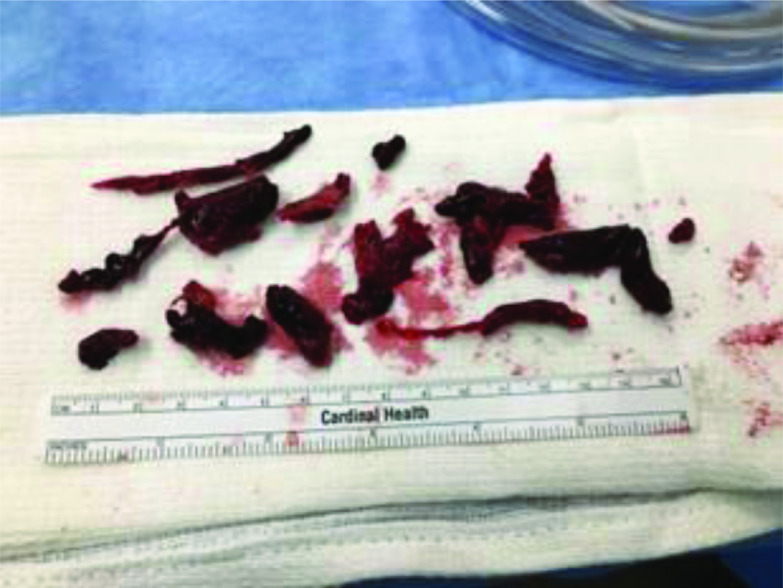
Thrombi directly extracted from the pulmonary arteries during open surgical embolectomy.

A 71-year-old male with a history of coronary disease, atrial fibrillation, obstructive pulmonary disease, and multiple prior lower extremity deep vein thrombosis (DVT) presented with syncope and acute shortness of breath. In the preceding month, the patient’s systemic anticoagulation with warfarin was held following a car accident with resultant subarachnoid hemorrhage (SAH). CT imaging demonstrated saddle PE with signs of RV strain. Systemic anticoagulation with heparin was initiated, but due to his recent SAH, systemic or catheter-based fibrinolytic therapies were contraindicated. Inferior vena cava (IVC) filter placement had been attempted but was aborted due to increasing respiratory distress. At this point, the growing clinical instability prompted cardiac surgery consultation, and emergent surgical pulmonary embolectomy was deemed appropriate. Intraoperative transesophageal echocardiogram (TEE) demonstrated a severely dilated RV, leftward bowing of the interventricular septum, and hyperdynamic left ventricular function. After performing a median sternotomy and giving standard heparinization, a 20-French (Fr) cannula was placed in the patient’s ascending aorta. The superior and inferior vena cava was cannulated with 28Fr and 32Fr cannulas respectively. Once cannulated and connected to the cardiopulmonary bypass (CPB) circuit, extracorporeal circulation was initiated. A 14-gauge antegrade needle was placed in the ascending aorta proximal to the arterial cannula for the administration of micro-blood cardioplegia and the perfusionist began to lower the patient’s core temperature to 32 °C. Once all the team members confirmed patient stability, the aorta was cross-clamped and the heart was arrested. Caval snares were tightened to optimize the unloading of the RV and create a bloodless field. A 20-Fr left ventricular (LV) vent was placed in the Right Superior Pulmonary Vein (RSVP) for decompression and collection of bronchial flow. The pulmonary artery (PA) was then transected and large amounts of thrombus were removed from both the left and right PA using direct extraction and Fogarty catheters (Fig. 1). After the removal of thrombi, a brief retrograde pulmonary perfusion and flush were performed utilizing the CPB circuit and retrograde placement of the original LV vent cannula in the RSVP, using techniques previously described [8]. Tubing that had previously been connected to the antegrade catheter for cardioplegia administration was placed on the retrograde “flush” catheter. After flushing the cardioplegia circuit of any hyperkalemic blood, we adjusted the CPB circuit to flow normothermic, patient autologous blood through the native cardioplegia circuit for retrograde flushing of the pulmonary system. Flow through the neo flush line was approximately 250 mL/min as limited by catheter size for the duration of 10 min, while we observed the open PA for additional emboli. The pulmonary arteriotomy was closed and the patient was weaned from cardiopulmonary bypass with moderate support for the RV. Extubation occurred on postoperative day (POD) 2. Anticoagulation was resumed with warfarin once the patient was extubated. The patient’s remaining hospital course was unremarkable, with discharge to a rehabilitation facility on POD 8.

Only mild RV dysfunction was present on the discharge echocardiogram.

## Case 2

**Figure 2:**
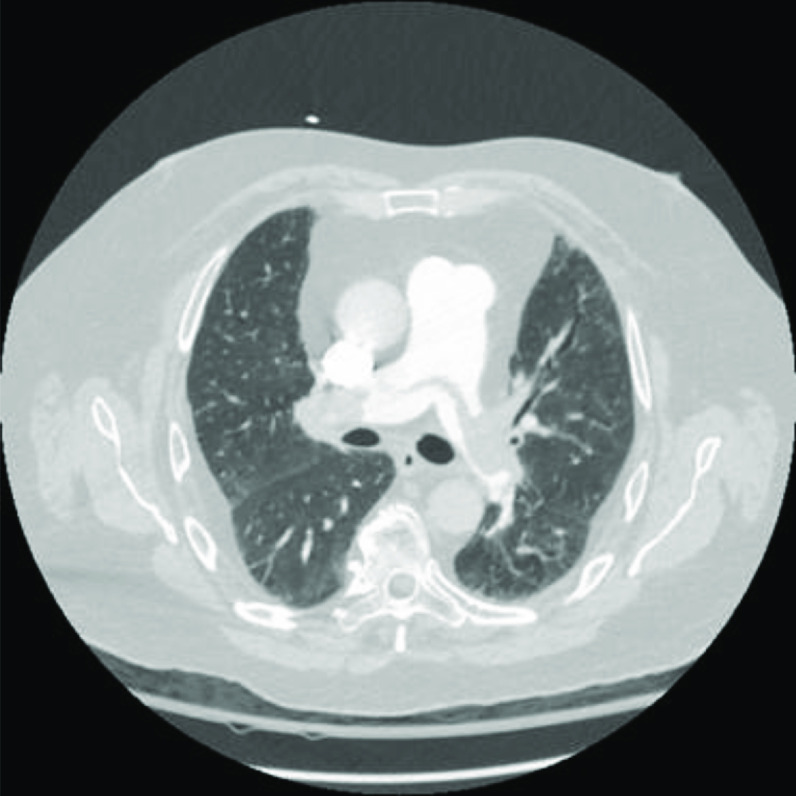
Chest CT demonstrating a saddle embolus with large volume bilateral central pulmonary emboli.

A 67-year-old female with a high-grade serous ovarian carcinoma stage IIB underwent major abdominal resection, and port placement for anticipated chemotherapy. On POD 10, an anastomotic leak with significant intra-abdominal sepsis prompted exploratory laparotomy with colostomy formation. Intraoperatively, she developed an acute drop in oxygen saturation. CT imaging revealed PE in branched vessels, in addition to superior vena cava (SVC) thrombus that was associated with her port catheter (Fig. 2).

Echocardiogram demonstrated a 2.2 × 1.5 cm mass at her port tip that was adherent to the lateral wall of the SVC/Right atrium (RA) junction. Given her current PE, risk for additional embolization of the thrombus associated with her port, and infection of her port and the associated thrombus in the setting of significant intra-abdominal sepsis, cardiac surgery consultation was obtained to help manage the removal of the port and associated thrombus. Catheter-directed aspiration-thrombectomy in conjunction with port removal was deemed an appropriate treatment option, as thrombolytic therapy was contraindicated given her recent major abdominal operations.

Once in the operating room, the patient was intubated and prepared for veno-venous bypass. The right and left femoral veins were accessed using ultrasound guidance. The right femoral vein was dilated sequentially, and a Gore Dry-Seal Flex Introducer Sheath (W.L. Gore & Associates, Inc, Newark, DE) was placed. Heparin was given for therapeutic effect at 5 U/kg, resulting in an activated clotting time of >200 s. The AngioVac cannula aspiration device was advanced through the sheath and into the RA using fluoroscopy and TEE guidance. The system consists of a 22 Fr suction catheter connected to a precrystalloid primed extracorporeal circuit containing a Terumo centrifugal head (pump) and the kit included a 120-micron filter, used to capture any entrained emboli. To reinfuse blood to the patient a 16 Fr cannula was placed in the left femoral vein. Veno-veno circulation was initiated by increasing the RPMs on the pump motor, causing the centrifugal pump to spin at a controlled rate. As the RPMs increase, negative pressure is generated in the central portion of the pump causing the patient’s blood and ideally the emboli to enter the circuit from the catheter in the right femoral vein placed proximal to the RA. Once the blood and intravascular debris have entered the circuit, all components flow through the 120-micron filter, trapping thrombus and only allowing small particles (red blood cells and other blood components) to flow past the filter, through the pump, and back into the patient via the reinfusion catheter inserted in the left femoral vein. In our specific case, the flow was gradually increased to 4 L/min. Anticoagulation status, hemodynamic stability, visualization of emboli entrapped in the filter, and venous line “chatter” are all monitored closely. Excessive suction may lead to hemolysis or even air cavitation in extreme cases, as the circuit may not contain any pressure monitoring. Notably, is not uncommon to use multiple filters as they may become impacted with embolic debris, disrupting flow through the circuit. In total, from initiation to termination of extracorporeal AngioVac flow, the run time lasted approximately 46 min.

**Figure 3:**
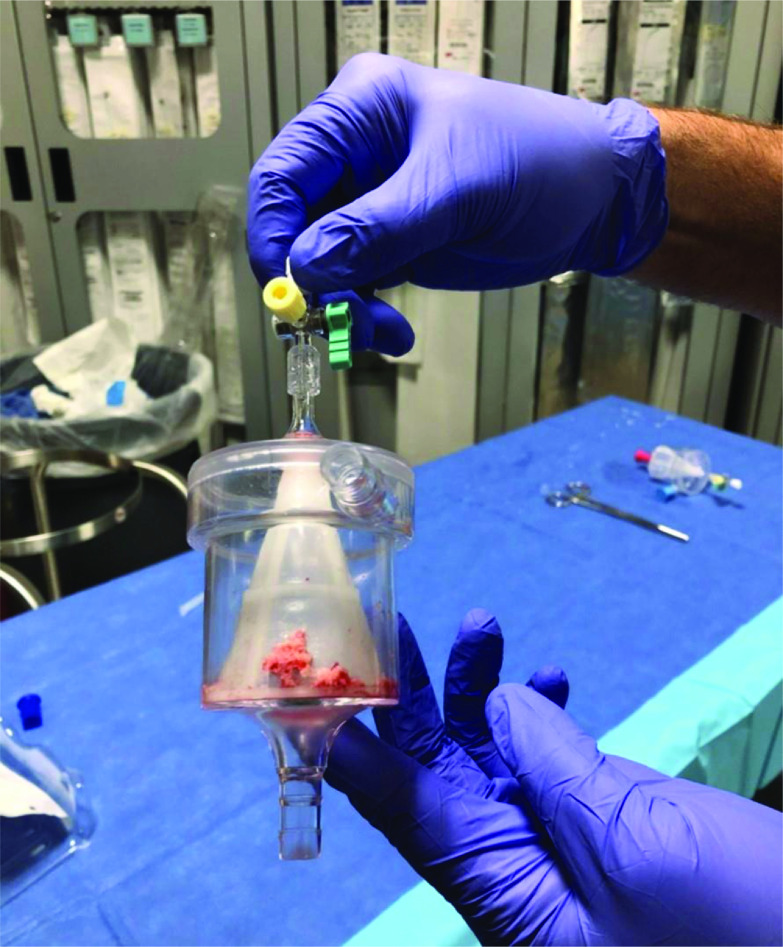
Captured debris from suction aspiration catheter.

Mechanical/rotational thrombolysis was performed by accessing the pre-existing port site and inserting the CLEANER system (Argon systems, Frisco CA) here to assist in mechanical lysis of the SVC/RA thrombus. Using a combination of direct aspiration from the AngioVac system and mechanical thrombolysis, a large amount of thrombus was debrided (Fig. 3), and the port was removed successfully. There was a brief drop in oxygen saturation during this process, but the patient was extubated successfully at the conclusion of the case and ultimately discharged from the hospital.

## Discussion

Treatment for intracardiac thrombi and pulmonary emboli should involve careful consideration of individual risks and benefits of therapy [1]. Important predictors of death in patients who are not candidates for systemic thrombolysis include shock, cancer, age >75 years, renal failure, and congestive heart failure [5]. The incidence of associated clot-in-transit with pulmonary embolus is approximately 418%, with mortality estimated between 25% and 40% [9].

Growing data suggest that specific high-risk patients with massive or submassive PE and/or clot in transit may have both survival and symptomatic benefit from advanced therapies in comparison to anticoagulation alone [1, 3–7, 9–10]. Long-term data for catheter-related therapies are lacking and mostly based on smaller case series [7]. Contemporary treatment algorithms include all of the following based on risk stratification: anticoagulation alone, thrombolysis, catheter-directed therapies, ECMO, and open surgical thrombectomy [1, 7].

Intracardiac thrombi typically present as non-elective, high-risk cases. As illustrated in these two cases managed by the same cardiothoracic surgery team, occurring in tandem and less than 24 h apart, the modern cardiothoracic surgical team must be facile with both minimally and maximally invasive therapies. Further investigation is needed to define more robust clinical guidelines for both traditional and novel treatment options in the management of intra-cardiac thrombus.

## Data Availability

All available data are incorporated into the article.
